# Asbestos and Intrahepatic Cholangiocarcinoma

**DOI:** 10.3390/cells9020421

**Published:** 2020-02-12

**Authors:** Giovanni Brandi, Simona Tavolari

**Affiliations:** 1 Department of Experimental, Diagnostic and Specialty Medicine, S. Orsola-Malpighi University Hospital, 40138 Bologna, Italy; 2 Center for Applied Biomedical Research, S. Orsola-Malpighi University Hospital, 40138 Bologna, Italy; simona.tavolari@unibo.it

**Keywords:** intrahepatic cholangiocarcinoma, asbestos, hepatic stem/progenitor cells

## Abstract

The link between asbestos exposure and the onset of thoracic malignancies is well established. However epidemiological studies have provided evidences that asbestos may be also involved in the development of gastrointestinal tumors, including intrahepatic cholangiocarcinoma (ICC). In line with this observation, asbestos fibers have been detected in the liver of patients with ICC. Although the exact mechanism still remains unknown, the presence of asbestos fibers in the liver could be explained in the light of their translocation pathway following ingestion/inhalation. In the liver, thin and long asbestos fibers could remain trapped in the smaller bile ducts, particularly in the stem cell niche of the canals of Hering, and exerting their carcinogenic effect for a long time, thus inducing hepatic stem/progenitor cells (HpSCs) malignant transformation. In this scenario, chronic liver damage induced by asbestos fibers over the years could be seen as a classic model of stem cell-derived carcinogenesis, where HpSC malignant transformation represents the first step of this process. This phenomenon could explain the recent epidemiological findings, where asbestos exposure seems mainly involved in ICC, rather than extrahepatic cholangiocarcinoma, development.

## 1. Introduction

Cholangiocarcinoma (CC) encompasses a heterogeneous group of malignancies developing from the biliary epithelial tree within (ICC) and outside (ECC) the liver [1]. In the past three decades a progressive increase in ICC incidence has been registered worldwide, while ECC appears stable or slightly decreasing [2]. Notably, ICC increase seems to have not reached a plateau and, basing on the global epidemiological trend, it has been estimated that about 50% of primary liver cancer deaths will be ascribable to this disease within 2035 [3]. The wide geographic variations between ICC and ECC incidence is thought to reflect a different distribution of host genetic and local risk factors. Currently some pathological conditions, such as primary sclerosing cholangitis, hepatolithiasis, bile duct cysts, Caroli’s disease, liver fluke infections and non-alcoholic steatohepatitis (NASH) have been recognized as risk factors for ICC (Table 1) [2]; however in Western countries the aetiology of about 50% of diagnosed ICCs still remains unknown. This observation, along with the wide molecular heterogeneity of this disease [4], strongly suggests that other risk factors may be involved in ICC development and in its global increase of incidence. Among the emerging risk factors, recent epidemiological studies have provided compelling evidences about a link between asbestos exposure and ICC.

On this basis, the present review summarizes the current epidemiological knowledge about asbestos and ICC development. The carcinogenetic mechanisms of asbestos, along with the translocation pathway of fibers to the biliary tree and their potential target cells in the liver are also discussed.

## 2. Asbestos Carcinogenesis

**Figure 1:**
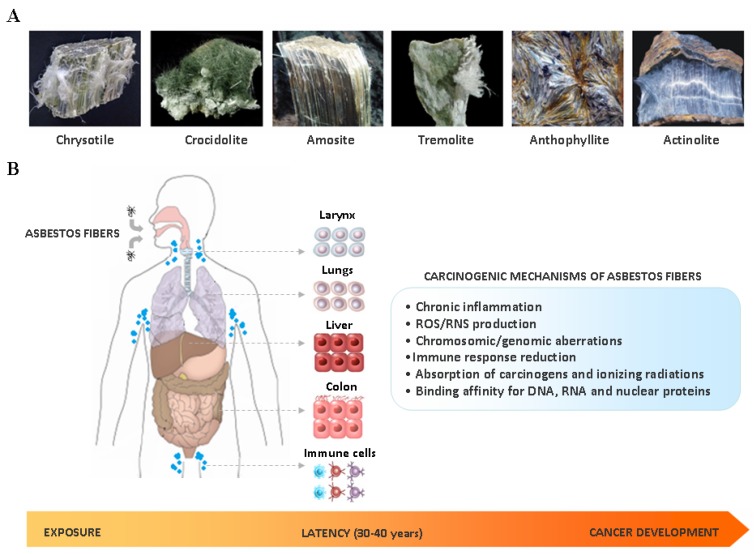
(**A**) Main types and chemical structure of asbestos fibers: chrysotile, belonging to the serpentine group, and actinolite, amosite, anthophyllite, crocidolite and tremolite, belonging to the amphibole group; (**B**) Inlated or ingested asbestos fibers may target the cells of different organs, including larynx, lungs, liver, colon and immune system. During the very long latency period of asbestos carcinogensis (30–40 years), cell malignant transformation may occur by a complex interplay among different mechanisms, including: chronic inflammation, reactive oxygen species (ROS)/reactive nitrogen species (RNS) production, induction of chromosomic/genomic aberrations, immune response reduction, absorption of carcinogens and ionizing radiations, and binding to nucleic acids and nuclear proteins.

The term asbestos is generically assigned to a group of naturally occurring fibrous silicates that have been largely employed in manufacturing and construction during the past century, due to their heat and chemical resistance, high mechanical and thermal stability and low cost. According to the chemical composition and crystalline structures, asbestos fibers can be divided into two groups: serpentines and amphiboles (Figure 1A). Chrysotile (also known as ‘white asbestos’) is the only member of the serpentine group and consists of an octahedral magnesium hydroxide layer intercalated between silicate tetrahedral layer; fibers are curled, flexible, long and easily breakable [5]. Currently it accounts for up to 90%–95% of commercially-used asbestos worldwide [6]. Amphiboles are all hydrated silicates and have double tetrahedral chains with Si_8_O_22_ composition distinguished from one another by the number of the cations Ca, Fe, Mg and Na that they contain [5]. Actinolite, amosite, anthophyllite, crocidolite and tremolite (also known as ‘brown asbestos’) belong to the amphibole group: fibers are rigid, short, sharp and highly resistant to chemical and biological solutions, and have a greater biopersistence compared to chrysotile [5,7].

The International Agency for Research on Cancer (IARC) classifies asbestos as a Group 1 human carcinogen [8]. Several epidemiological and molecular studies have provided strong evidences that asbestos-induced carcinogenesis is a complex event resulting from different causative factors (Figure 1B), including the specific physicochemical characteristics of the fibers (dimension, surface reactivity and chemical composition), time and dose of exposure, and finally host genetic determinants.

Fiber dimension represents one of the main properties determining asbestos toxicity. Thin and long fibers may induce a state of chronic inflammation in target tissues, due to their ability to persist for a very long time in human body. Indeed as macrophages may reach 10–20 μm diameter, fibers longer 20 μm cannot be completely phagocytosed, thus leading to incomplete or ‘frustrated’ phagocytosis, characterized by the prolonged release of pro-inflammatory cytokines by activated macrophages [9]. Asbestos-induced chronic inflammation may activate multiple signaling cascades involved in cell proliferation and survival, including the epidermal growth factor receptor (EGFR) pathway [10]. Moreover sustained inflammatory signals may cause alterations in the cellular epigenetic program and induce gene hypermethylation [11]. In line with this observation, epigenetic silencing of CDKN2A gene, that encodes the tumor suppressors p16(INK4A) and p14(ARF), has been reported as an early and key molecular event occurring during the latency period between exposure to long asbestos fibers and cell malignant transformation [12]. Chronic inflammation generated by the prolonged phagocytic activity in order to eliminate biopersistent fibers also induce the release of reactive oxygen species (ROS) and reactive nitrogen species (RNS) by activated macrophages [10]. ROS and RNS are known to promote cell malignant transformation by induction of DNA single/double strand breaks, DNA base modifications, formation of DNA adducts, lipid peroxidation and activation of signalling cascades involved in cell proliferation and survival [10]. ROS and RNS release in target tissues in turn recruit other macrophages and inflammatory cells at the sites of fiber deposition, thus sustaining the prolonged production of free radicals and chronic inflammation [10]. Asbestos fibers may promote ROS and RNS production also via Fenton reaction, as some types of amphiboles contain iron as integral components of their chemical structure, whereas other types of fibers as surface impurity [13]; notably, crocidolite and amosite fibers are particularly iron-rich, containing 20%–30% iron by weight. Moreover fibers longer than 8 μm, especially amphibole, can become coated with iron rich proteins, such as ferritin and hemosiderin, thus favoring ROS production [14].

Asbestos fibers may also absorb on their surface ionizing radiations and different types of carcinogens, leading to their accumulation in target cell [15]; in particular, benzo(a)pyrene has high affinity for asbestos fibers and a cooperative mutagenic effect has been reported [16].

Another mechanisms linked to asbestos carcinogenesis is linked to the ability of fibers, especially chrysotile and crocidolite, to physically interact with chromosomes and mitotic spindle of dividing cells, resulting in multipolar mitosis and numerical (aneuploidy, polyploidy and hyperploidy) and structural (deletions, translocations, inversions, duplications and non-disjunctions) chromosomal alterations [10,17,18]. Previous studies have shown that chrysotile and crocidolite fibers can also directly interact with chromatin-binding proteins and histones, respectively, thus affecting chromatin structure [19]; furthermore, chrysotile may interfere with mRNA transcription and protein translation by binding to RNA-binding proteins [19].

Asbestos fibers may also target immunocompetent cells, leading to a decrease of tumor immunity by enhancement of regulatory T cell function, reduction of CXCR3 chemokine receptor expression in CD4+ T helper cells, and impairment of killing activities of CD8+ lymphocytes and NK cells [20].

Host genetic background may play an important role in influencing cancer susceptibility in asbestos-exposed individuals. Germline mutations of the BRCA1 Associated Protein 1 (BAP1), the only gene that has been proposed to influence environmental carcinogenesis, have been indeed reported to increase cancer risk after minimal exposure to asbestos fibers in animal models [21]. In particular BAP1 (+/-) mice exposed to low doses of asbestos developed malignant pleural mesothelioma (MPM) at a similar rate of BAP1+/+ mice exposed to 10 times higher doses [21]. Furthermore, MPMs developed in BAP1 (+/-) mice have been found to arise faster than in wild-type, showing an increased invasiveness and proliferation rate [22]. Drawing parallels to humans these findings suggest that, compared to wild-type subjects, subjects carrying BAP1 germline mutations may be more susceptible to asbestos carcinogenesis, even when exposed to low levels of fibers. In line with this hypothesis, we recently reported the case of a patient carrying a BAP1 germline mutation and exposed to low levels of asbestos, who developed an ICC at a young age (47 years-old) [23].

Although banned in 52 countries (including all European Union member countries), environmental and occupational exposure to asbestos still represents a serious global health problem. Currently 125 million people worldwide are exposed to this compound [8], and even in countries banning its use since the early 1990s the number of asbestos-related diseases is rising [8]. Indeed it has been estimated that if global use of asbestos were to cease today, a decrease in the incidence of asbestos-related diseases would become evident in approximately 20 years [24]. Since the latency period between exposure and disease development may be many decades (30–40 years), it is expected that the growth rate of asbestos-related cancers will increase in the coming years [8].

At present, the link between asbestos exposure and the development of MPM and lung cancer is well defined and widely accepted by the international community. Epidemiological evidences clearly suggest that asbestos fibers may be also implicated in the development of extra-pulmonary malignancies, especially of the gastrointestinal tract (GI), including ICC [25,26,27,28,29]. However, why some individuals exposed to asbestos preferentially develop GI tumors rather than thoracic malignancies is still unknown and the question remains open.

## 3. Epidemiological Evidences about Asbestos Exposure and ICC Development

**Figure 2:**
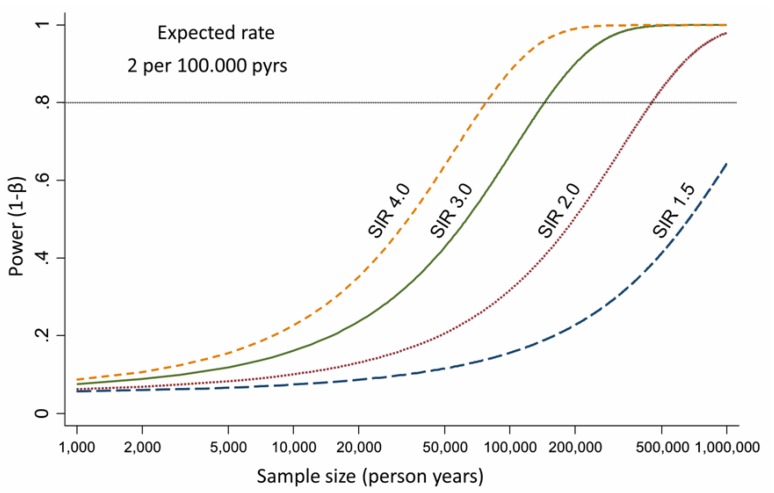
Statistical power for the study of ICC and asbestos exposure calculated under several scenarios according to Armstrong [32]. SIR: standardized incidence ratio.

Currently the amount of epidemiological studies on ICC incidence and asbestos exposure is limited. Despite this possible association has been suggested in some cohort studies (Table 2), most of them reported estimates referred to the broad category of primitive liver cancers, without specific data on ICC. The lack in these studies of specific data on ICC development and asbestos exposure may be ascribable to different causes. The first important issue when interpreting reported epidemiological data on CC is the evolving WHO International Classification of Disease (ICD) coding system, which is internationally used by cancer registries to record different cancers. Indeed, this coding system includes a specific code for ICC only from version 8 (the ICD-7 code 155.0 included all forms of liver cancer). A second important issue regards possible misclassification, as some ICCs may be misdiagnosed as cancers of unknown primary (CUPs), hepatocellular carcinomas (HCCs) or mixed HCC-ICCs [30]. Moreover, although showing an increase in global incidence, ICC still represents a minority of primary liver cancers [31]; this fact as two important consequences. First, relative risks estimated for liver cancers are driven by the vast majority of HCCs, with ICCs playing a minor role. Secondly, only extremely large cohorts (as those based on nation-wide registers) have sufficient statistical power to capture a clear relationship between a specific risk factor and the development of a disease with low incidence. Figure 2 shows the statistical power for the study of ICC under several scenarios according to Armstrong [32]. In case of a link between asbestos and ICC, assuming a baseline incidence of 2 per 100,000 person-years (higher than the actual incidence documented in Europe in 2007 [33]), 450,000 person-years should be studied to observe a standardized incidence rate (SIR) of 2.0 with a statistical power of 80%. Furthermore, it should be underlined that the SIRs, calculated with reference to the entire population, are usually lower than the relative risks estimated in case-control studies (or in cohort studies including a comparison group of unexposed subjects). Although several studies showing data on occupational cohorts exposed to asbestos have been published, on the balance, it is not surprising that an increased risk of ICC due to asbestos exposure has been seldom reported in scientific literature. Indeed, the vast majority of the cohorts did not provide the statistical power and the diagnostic information needed to study ICC.

Recently, two different case-control studies highlighted the role of asbestos in ICC development. The first study was based on historical data from 69 ICC and 86 ECC cases occurring at S. Orsola-Malpighi University Hospital of Bologna (Italy) between 2006 and 2010 [28]. The cases were individually matched by calendar period of birth, sex, and region of residence to historical hospital and population controls. Occupational exposure to asbestos was retrospectively assessed considering job titles obtained from work histories. An OR = 4.81 (95% CI 1.73–13.33) for ICC risk was reported among subjects occupationally exposed to asbestos for over 30 years, whereas a limited evidence was observed for ECC (OR = 2.09, 95% CI 0.83–5.27). These findings have been confirmed in a case-control population-based study nested in the Nordic Occupational Cancer cohort (NOCCA) cohort [29]. In this study 1458 ICC and 3972 ECC cases occurring in Finland, Iceland, Norway and Sweden starting from January 1920 were analyzed. Each case was individually matched by birth year, sex, and country to five population controls. The cumulative exposure to asbestos [measured in fibers (f)/mL × year] was assessed applying the NOCCA job exposure matrix to data on occupations collected during national population censuses (conducted in 1960, 1970, 1980–1981, and 1990). An increased risk for ICC, but not for ECC, was observed by cumulative exposure to asbestos: 0.1–4.9 f/mL × years, OR = 1.1 (95% CI 0.9–1.3); 5.0–9.9 f/mL × years, OR = 1.3 (95% CI 0.9–2.1); 10.0–14.9 f/mL × years, OR = 1.6 (95% CI 1.0–2.5); ≥15.0 f/mL × years, OR = 1.7 (95% CI 1.1–2.6). Overall these two studies suggest that exposure to asbestos may represent a risk factor for ICC development; conversely the association with ECC seems null or weak.

To provide further evidence about an association between asbestos exposure and ICC development, we recently conducted a prospective case-control study (Cholangiocarcinoma Aetiology: Role of Asbestos, CARA study) in collaboration with the Occupational medicine Unit of S. Orsola-Malpighi University Hospital of Bologna (Italy). The results obtained have been shown at the II biennal congress of the European Network for the Study of Cholangiocarcinoma (ENS-CCA) (Rome, 2018). A total of 168 CC cases (116 ICCs and 52 ECCs) and 185 controls (inpatients referring to our hospital for non-neoplastic diseases) were recruited. Data on established or suspected CC risk factors were extracted from the clinical records of the cases and controls. Exposure to asbestos was based on categories derived from the ReNAM questionnaire categorization: unlikely (reference category), possible and likely. The results obtained in this study on incident cases not only confirm the results obtained in the two studies on prevalent cases [28,29], but even reinforce the link between asbestos exposure and ICC risk. Moreover, it is worth to underline that in our clinical experience more than 40% of ICC patients who are diagnosed in absence of any known risk factors for this disease result exposed to asbestos according to ReNaM questionnaire. This observation suggests that among the ethiologic factors linked to ICC development, asbestos is likely one of most responsible for ICC increasing incidence worldwide, at least in Western countries.

Basing on these findings, in order to identify putative molecular biomarkers of asbestos exposure in ICC patients, we performed next generation sequencing analysis in patients exposed and not-exposed to asbestos (EtherBil study, NCT02184871). Notably, patients exposed were found to display a distinctive molecular profile compared to the group of not exposed. These findings are in line with some studies in lung tumors showing a different molecular profile between asbestos-exposed and non-exposed patients, suggesting that asbestos could induce typical molecular alterations in target cells [48,49,50,51].

## 4. Adult Liver, Biliary Tree and Stem Cell Niches

In continually renewing tissues, such as intestinal mucosa and epidermis, tissue homeostasis and regeneration after injury is sustained by the dynamic activity of resident stem cells, that are endowed with the ability of long-term self-renewal and multi-lineage differentiation [52]. Stem cell reside in the stem cell niche, a specialized tissue microenvironment comprising different cell types, extracellular matrix, growth factors and cytokines released by the cells of the niche; the fine interaction among all of these components ultimately controls stem cell fate [53].

In the liver, tissue homeostasis and regeneration after injury is more complex, due to the unique characteristics of this organ. Indeed, in normal conditions the liver is silent organ, with a very slow turnover of hepatocytes and cholangiocytes. However, after a mild/moderate injury, a regenerative response rapidly occurs, quickly restoring the pristine liver parenchyma [54]. This efficient regenerative response is driven by mature hepatocytes and cholangiocytes, that leave the G_0_ quiescent state and enter the cell cycle and mitosis [54].

A different scenario occurs when the liver parenchyma is subjected to acute or chronic injury. In these circumstances, the regenerative capability of hepatocytes and cholangiocytes is lost or significantly impaired, due to necrosis/apoptosis or replicative senescence, this last resulting from the continuous turnover over 20–30 years of chronic liver disease, that gradually leads to telomere shortening in these cells [55,56]. Necrosis/apoptosis and senescence of liver mature epithelial cells lead to the activation of the so-called “ductular reaction”, characterized by the appearance of reactive ductules, anastomosing strands comprising transit amplifying cells committed towards hepatocyte and/or cholangiocyte lineages, depending on the cell type mostly damaged in the liver [57]. Compelling evidences have shown that ductular reaction is driven by the activation of the stem compartments within the liver and along the biliary tree [58]. Currently two different stem cell niches have been described in the adult liver and the biliary tree: the canals of Hering and the peribiliary glands (PBGs) [59].

The canals of Hering are adult remnants of the ductal plate of foetal and neonatal liver and are localized in the portal tract and periportal parenchyma. These canals, whose lumen is partially lined with hepatocytes and cholangiocytes, are the most peripheral branches of the biliary tree and represent the anatomical and physiologic link between hepatocyte canaliculi and biliary duct systems. The canals of Hering contain human hepatic stem/progenitor cells (HpSCs), bipotent progenitor cells capable to differentiate into mature hepatocytes and cholangiocytes [60]. Commitment of HpSCs towards mature hepatocytes or cholangiocytes is characterized by the appearance of a trans-amplifying population with an intermediate phenotype expressing variable mixtures of the phenotypic traits of fully differentiated cells and their precursors [61]. The underlying mechanisms driving HpSC differentiation towards committed lineages is complex and not fully clarified. Recent advances suggest that it depends on an intimate interplay between HpSCs and the factors released by the other cell types of the stem niche, including portal myofibroblasts, hepatic stellate cells and resident macrophages (Kupffer cells) [61].

PBGs are tubulo-alveolar glands located in the deeper tissue of bile duct walls and communicating with the duct lumen. They are distributed along the biliary tree and supply the renewal of the epithelium of large intrahepatic bile ducts and extrahepatic biliary tree [62]. PBGs contain biliary tree stem/progenitor cells (BTSCs), precursors of endodermal origin able to differentiate toward hepatocytes, cholangiocytes and pancreatic islets [63,64]. BTSCs are mostly located at the bottom of PBGs, while their descendants undergoing lineage maturation are localized at the neck, in close contact with the surface epithelium. Recent findings have shown that PBGs connect directly into the canals of Hering, making a continuous network of stem cells that sustain the renewal of the liver and biliary tree [62].

The activation of resident stem/progenitor cell compartments within the liver and the biliary tree during chronic injury, along with the tight association between chronic liver damage and the development of primitive liver cancers, strongly supports the notion that primitive liver cancers arising from chronic injury may have a stem/progenitor cell origin. Due to the inherent capability of self-renewal and longevity, that allow sequential accumulation of genetic mutations over the years, stem cells represent indeed the ideal target for neoplastic transformation triggered by chronic injury. The putative stem/progenitor cell origin of some primitive liver cancers is also reinforced by the existence of poorly differentiated combined HCC-ICC, expressing a mixture of phenotypical traits belonging to hepatocytes, cholangiocytes and progenitor cells [65].

## 5. Asbestos Fibers in the Liver and the Biliary Tract

The prerequisite for a role of asbestos in ICC carcinogenesis is the presence of fibers in the biliary tree within the liver. The first study reporting the detection of asbestos fibers in the biliary tract refers to a patient with asbestosis; post-mortem examination revealed a cancer arising from the cystic duct with short asbestos bodies and fragments with a similar shape of those observed in the lung [66]. More recently, the deposition of asbestos fibers in the biliary tract has been confirmed by some studies of Grosso et al. [67,68,69,70]. In the first explorative study, five patients living in Casale Monferrato (a well-known asbestos-polluted Italian city) undergone surgery for gallbladder stones were analyzed [67]. In three out of five patients, mineral phases consistent with chrysotile fibers were detected in the gallbladder. Moreover fibers ascribable to asbestos, even if not belonging to the serpentine group, were identified in the bile of the fourth patient. Only in the fifth patient, a 13-year-old child, no fibers were detected. However this finding may be consistent with the hypothesis that asbestos fibers require a long time to translocate to extra-pulmonary organs [71]. A high concentration of fibers has been also detected in the gallbladders of patients environmentally/occupationally exposed to asbestos, who suffered of severe bile tract diseases and died of MPM [68,69]. The presence of fibers/bundles of chrysotile has been also detected in five out of seven patients (71%) from Casale Monferrato affected by ICC; fibers incorporation occurred at the boundary between the healthy and neoplastic hepatic tissue or in fibrocollagen tissue produced by the neoplasia [70]. Notably, chrysotile was the only type of fibers observed in the liver, whereas in the gallbladder of patients with MPM also crocidolite was detected [68,69]; this observation is likely related to the easier translocation in extrapulmonary sites for serpentines, whose fibers are thinner than amphiboles [72].

**Figure 3:**
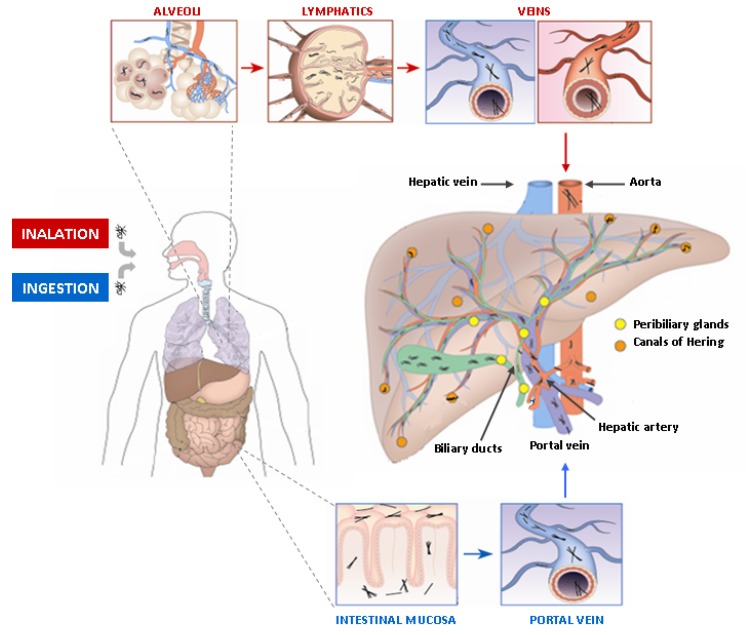
Translocation pathway of asbestos fibers in the body. Asbestos fibers are introduced into the body by inhalation and ingestion. Inhaled fibers can reach pulmonary alveoli, where they are drained by convective flows into pulmonary lymphatics. Once they reached veins through the lymphatic system, they can potentially reach all organs via the circulatory system, including the liver by the hepatic artery. Ingested fibers can across the intestinal mucosa and be finally delivered to the liver through the portal vein. In the liver and along the biliary tree two different stem cell niches have been described: the canals of Hering, containing hepatic stem/progenitor cells (HpSCs) and distributed along the most peripheral branches of the biliary tree, and the peribiliary glands, that contain biliary tree stem/progenitor cells. Peribiliary gland distribution starts from the septal/segmental bile ducts and ends extrahepatically in the hepatopancreatic common duct near the duodenum.

Although further studies on larger cohorts are needed to corroborate these preliminary results, overall these findings provide a proof of evidence that asbestos fibers can reach the biliary tract and the liver. Even if the exact mechanism still remains to be elucidated, the presence of these fibers in the biliary tree within the liver could be explained in the light of their translocation pathway (Figure 3). Asbestos fibers can be introduced into the body by two different mechanisms: inhalation (the most involved) and ingestion. In the inhalation pathway, once inhaled, fibers remain parallel to the airflow direction in the upper respiratory tract and reach pulmonary alveoli. Here, they can be drained by convective flows into pulmonary lymphatics that act as a pump [71]. The clearance of asbestos fibers from lung interstitium through the lymphatic system is a relatively slow phenomenon and, in fact, particles are eventually trapped in the tracheo-bronchial lymph nodes that become “reservoirs of retained material” [71]. Once reached veins through the lymphatic system, asbestos fibers can potentially reach all organs via the circulatory system, including the liver and the biliary tree by the hepatic artery. As to the ingestion pathway, it has been suggested that asbestos fibers can across the intestinal mucosa and be finally delivered to the liver through the portal vein [73].

**Figure 4:**
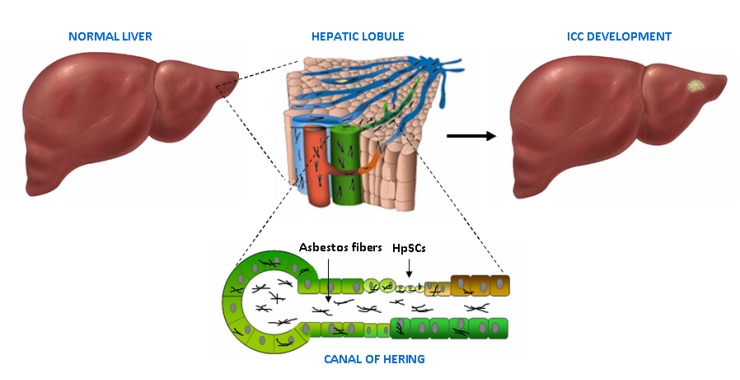
In the liver, asbestos fibers could remain trapped in the smaller bile ducts, particularly at the level of the canals of Hering, where they may exert their carcinogenic effect for a long time, inducing HpSC malignant transformation and finally ICC development.

The concentration of asbestos fibers in target tissues depends on the number of fibers that are trapped and cannot be removed. As to the liver, the high microvascular permeability of the hepatic sinusoids may facilitate fiber deposition [66]; moreover, Kupffer cells located within the hepatic parenchyma could exert a role similar to alveolar macrophages, currently considered pivotal actors in the carcinogenic process of asbestos-related lung cancer [74]. In the liver parenchyma, thin and long asbestos fibers could remain trapped in the smaller bile ducts, particularly at the level of the canals of Hering, thus inducing HpSC malignant transformation (Figure 4). In this scenario, the chronic liver damage induced by asbestos fibers over the years could be seen as a classic model of stem cell-derived carcinogenesis, where HpSC malignant transformation represents the first step of this process. This phenomenon could explain the recent epidemiological studies reporting a link between asbestos exposure and ICC, rather than ECC, development. Moreover, due to the ability of HpSC to differentiate towards both hepatocyte and cholangiocyte lineages, it is conceivable that exposure to asbestos could be also responsible for other primitive liver tumor development, such as hepatocellular carcinoma (HCC) and combined HCC-ICC; however this last hypothesis still remains to be investigated.

## 6. Conclusions

Although traditionally considered a rare tumor in Western countries, ICC incidence and mortality rate are rising worldwide and a further increment is expected in the near future, thus justifying the increasing scientific attention toward this malignancy. The most recent epidemiological studies have provided evidences about a link between asbestos exposure and ICC risk and may explain, at least in part, the global increase of this disease. Currently, the knowledge of the etiopathogenic and molecular mechanisms underlying ICC is rapidly evolving, also thanks to the availability of new and powerful analytical technologies with high throughput such as next-generation sequencing. The use of these methodologies has already made possible to identify a distinctive molecular signature in patients suffering from tumor diseases of environmental origin, and preliminary results confirm this finding also for ICC patients exposed to asbestos. As asbestos still represents a serious global health problem, the identification of molecular markers of exposure could not only improve the monitoring and the surveillance procedures for cohorts of subjects exposed and at high-risk of disease, but also allow the development of more personalized, stratified and efficacious strategies for the diagnosis, and perhaps treatment, of this dismal malignancy.

## Figures and Tables

**Table 1:** Risk factors for intrahepatic cholangiocarcinoma (ICC).

Risk Factor	Association with ICC
Bile duct cysts/Caroli’s disease	very strong
Primary sclerosing cholangitis/cholangitis	very strong *
Hepatolithiasis	strong/very strong
Cholelithiasis/choledocholithiasis	moderate/strong
Cirrhosis	strong/very strong
HBV/HCV infection	moderate/strong
Hemochromatosis	moderate
Inflammatory bowel disease/chronic pancreatitis	moderate
Duodenal/gastric ulcer	weak/modest
O. viverrini/C. sinensis infection	strong *
Diabete type II	weak/modest
Obesity	weak/modest *
NAFLD/NASH	strong
Alcohol	moderate
Cigarette smoking	weak/modest
Thorotrast	very strong *
1,2-dichloropropane	very strong *

Abbreviations: HBV, hepatitis B virus; HCV, hepatitis C virus; NAFLD, non-alcoholic fatty liver disease; NASH, non-alcoholic steatohepatitis. Weak/modest association (OR: 1–1.7); moderate association (OR: 1.7–3); strong association (OR: 3–8); very strong association (OR > 8). * Available studies did not distinguish between ICC and extrahepatic cholangiocarcinoma (ECC).

**Table 2:** Asbestos and liver/biliary tract cancer in cohort studies.

Reference	Period	Cohort	Workers’ Category	SMR or SIR * (95% CI)	Tumor Site
Selikoff I. et al., 1991 [34]	1967–1987	17800 (M)	Insulator workers	1.08 2.61	Liver Bile ducts + Gallbladder
Battista G. 1999 [35]	1945–1970	734 (M)	Railway workers	241 (126–420)	Liver
Berry G. et al., 2000 [36]	1933–1980	5000 (M/F)	Factory workers	2.66 (1.28–4.89)	Liver + Bile ducts + Gallbladder
Wingren G. 2004 [37]	1964–1997	1229 (M/F)	Art glassworks	* 2.00 (0.41–5.84) (M) * 4.35 (0,75–10.59) (F)	Liver + Bile ducts
Hein MJ. et al., 2007 [38]	1940–2001	3072 (M/F)	Textile workers	1.05 (0.51–1.94)	Liver + Biliary tract
Pira E. et al., 2007 [39]	1946–1984	1966 (M/F)	Textile workers	237 (118–425)	Liver
Clin B. et al., 2009 [40]	1978–2004	2024 (M/F)	Textile workers	* 1.61 (0.86–2.75) * 1.92 (0.38–5.6)	Liver Biliary tract
Wang X. et al., 2013 [41]	1972–2008	586 (M) 272 (F)	Textile workers	1.34 (0.81–2.21) –	Liver + Bile duct
Hogstedt T. et al., 2013 [42]	1958–2006	6320 (M/F)	Chimney sweeps	* 2.48 (1.47–3.91) * 1.6 (0.19–5.78)	Liver Bile ducts
Boulanger M. et al., 2015 [43]	1978–2009	2024 (M/F)	Textile workers	* 1.85 (1.09–2.92) (M) * 2.84 (0.76–7.26) (M)	Liver Biliary tract
				* 1.85 (1.09–2.92) (F) * 2.84 (0.76–7.26) (F)	Liver Biliary tract
Wu W. et al., 2015 [44]	1975–1989	4427 (M/F)	Shipbreaking workers	1.6 (1.08–2.36)	Liver + Intrahepatic bile ducts
Pira E. et al., 2016 [45]	1946–2013	1977 (M/F)	Textile workers	1.06 (0.55–1.86)	Liver
Pira E. et al., 2017 [46]	1946–2014	1056 (M)	Miners	0,65 (0.21–1.51)	Liver
Luberto F. et al., 2019 [47]	1934–2006	13076 (M/F)	Cement workers	0.99 (0.81–1.20) (M) 0.84 (0.42–1.60) (F)	Liver + Intrahepatic bile ducts

Abbreviations: M: males; F: females; SMR: standardized mortality ratio; SIR: standardized incidence ratio. * Studies reporting SIR (standardized incidence ratio) and not SMR (standardized mortality ratio).
